# Process, quality and challenges of diabetes care in primary care: a study of district health network in Thailand

**DOI:** 10.1017/S1463423620000468

**Published:** 2020-10-27

**Authors:** Jumnean Somanawat, Kritsanee Saramunee, Suratchada Chanasopon

**Affiliations:** 1 Master Programme of Pharmacy (Clinical Pharmacy), Faculty of Pharmacy, Mahasarakham University, Maha Sarakham, Thailand; 2 Social Pharmacy Research Unit, Faculty of Pharmacy, Mahasarakham University, Maha Sarakham, Thailand

**Keywords:** primary care, process, structure, type II diabetes care

## Abstract

This study aimed to describe the process of care, assess the quality of care based on defined indicators, and identify challenges associated with providing diabetes care via sub-district health promotion hospital (SHPH) facilities in Thailand. Primary care policy has directed that diabetes care be delivered via SHPH in order to reduce hospital congestion and minimize travel costs for patients. Limited data is available regarding the structure for providing care. Likewise, barriers to delivery of optimal care have not been well defined, especially from the perspective of health care providers. This study employed mixed-methods research, which included semi-structured interviews to gain insights into the current diabetes care process, a descriptive study to evaluate quality of care, and use of a focus group to identify challenges associated with delivery of diabetic care via SHPH. Diabetes care processes in primary care included multiple steps and involved collaboration between various health care providers at both the hospital and SHPH. Four process indicators and one outcome had been achieved but performance of other indicators was apparently low. Three factors were found to pose challenges to providing this service: the resources of the health service, the delivery of services, and patient factors. SHPH require additional support, particularly in the areas of primary care workforce, finance, medical device procurement, and patient information systems. While delivery of diabetes care via primary care centers has been well established in Thailand, regional differences in the quality of care persist. Additional support is required to strengthen the primary care system nationwide.

## Introduction

Diabetes mellitus has become a significant worldwide public health problem, with the number of patients steadily increasing each year. The global prevalence of diabetes among adults over 18 years of age has increased from 4.7% in 1980 to 8.5% in 2014, rising most rapidly in middle- and low-income countries (World Health Organization, 2017). In Thailand, a public health survey reported that the prevalence of diabetes in individuals of age 15 years and older had increased to 8.9% in 2014 (International Health Policy Program, 2016). Diabetes is a chronic health condition that requires long-term treatment. Thus, the burden associated with diabetes is high. This situation leads to increased expenditures for care provided by the national health system. In 2008, the cost of medical treatment for diabetes was 47 596 million baht (approximately 1534 million USD), equal to 28 207 baht (approximately 909 USD) per patient per annum (Institute of Medical Research and Technology Assessment, 2014).

Management of chronic disease, including diabetes, is a priority for primary health care facilities, especially in low- and middle-income countries (Beaglehole *et al.*, 2008). Since the primary focus of diabetes care is to prevent or delay complications, it is necessary for patients to obtain good quality care in order to achieve this goal (American Diabetes Association, 2016). Campbell and colleagues have defined the term ‘quality of care for the individual’ as ‘whether individuals can access the health structures and processes of care which they need and whether the care received is effective’. Three components are considered essential for high quality care: the structure of health care, the process of care, and the consequences of care (outcome) (Campbell *et al.*, 2000). In practice, the components of quality care are transformed into quality indicators that are used to encourage effective management of diabetes care. Such guidelines have often been used by local, national, and international organizations (Stone *et al.*, 2013) and policy makers (Campbell *et al.*, 2002).

Health service in Thailand is commissioned based on geographical areas: sub-district, district, province, and region. Primary care is delivered at the sub-district and district levels. A sub-district health promotion hospital (SHPH), installed in all sub-districts, may be the smallest health service offered, but it is the one that is established closest to a community. Within each district, SHPH work cooperatively with a district hospital to provide primary health care and other health services. Secondary and tertiary services are provided at the provincial and regional levels (Jongudomsuk *et al.*, 2015). The Ministry of Public Health in Thailand has implemented a national primary care policy by offering diabetes care at primary care centers, the aims being to reduce congestion at public hospitals and facilitate access to health service providers (Bureau of Non-communicable Diseases, 2017). This service has typically been provided via SHPH. To encourage the use and monitor performance of this service, a series of quality indicators for diabetes care have been described according to the recommendations of the Toward Clinical Excellence’ Network (TCEN). Fifteen indicators covering two dimensions have been employed: six process indicators and nine clinical indicators (Bureau of Non-communicable Diseases, 2017).

Two participatory action research studies previously conducted in Central (Sadtrakulwatana, 2018) and Southern Thailand (Samphawamana *et al.*, 2017) focused on designing appropriate models for diabetes care in primary care units. The diabetes care models proposed by these studies differed slightly, depending on local context in each case. However, the quality of care was not evaluated in either study. Two earlier studies had evaluated the quality of diabetes care by using process and clinical indicators (Parinyasakulwong, 2009; Rangsin *et al.*, 2012). Parinyasakulwong (2009) reported that diabetes care provided by primary care units was associated with a lower quality of care compared to the community hospital. For hospital-based settings, the quality of care according to process indicators was satisfactory, but while approximately 80% of patients had glycemic levels and HbA1c monitored regularly, only one-third achieved their glycemic goals (Rangsin *et al.*, 2012). Quantitative assessment of diabetes care in primary care settings is limited. Moreover, an evaluation of the structure of health care service from the perspective of the operational workers is lacking. Therefore, this study aimed to describe the process of care, assess the quality of care based on defined indicators, and identify any challenges to delivery of diabetes care via SHPH in Thailand.

## Methods

### Study design and setting

This study used a mixed-method research methodology composed of three steps: a semi-structured interview to gain insight into the current diabetes care process, a descriptive study to evaluate quality of care, and a focus group to identify challenges underlying diabetic care delivered via SHPH. This study was conducted in one district health network area in Northeast Thailand. Health services in this area included one community hospital (30 beds) located in the district center and 10 SHPHs spread around the district. The district health network boards established a policy agreement in which well-controlled diabetic patients are referred to a SHPH for care. This strategy was expected to increase the ease of access to care for patients and decrease the number of outpatient hospital visits.

### Step 1: semi-structured interview

The semi-structured interview was employed among primary care practitioners to obtain important information about the diabetes care process. This step focused on delivery of diabetes care at SHPH and evaluation of key resources supporting the care process. The key resources included policy, finance, health workforce, accessibility to essential medicines/technologies, and patient health information systems. Participants were also asked to explain the criteria used to refer patients to a SHPH or a hospital, describe how patients’ data were recorded, and delineate how the local community was involved in the implementation of this service. The interview guide was reviewed by two experts to ensure validity of the protocol. Ideally, interviews were conducted at the participant’s workplace by a trained interviewer (first author) and lasted approximately 30 min. Alternatively, a telephone interview was an option if a participant’s schedule did not permit an in-person meeting.

### Step 2: descriptive study

An electronic database of non-communicable diseases was reviewed to retrieve essential data related to diabetic patients. The inclusion criteria included patient’s age ≥ 18 years, diagnosis of type II diabetes by a physician, oral antidiabetic agent prescribed for patient, and patient continuously received care from a sub-district primary care facility during the 2015/2016 fiscal year (October 2015–September 2016). Verification of the quality of care for diabetes was based on recommendations established by the TCEN regarding process and clinical indicators. The process indicators included six items that encompassed laboratory tests or physical examination: HbA1c levels, lipid profile, microalbuminuria, administration of angiotensin converting enzyme (ACE) inhibitor, or angiotensin receptor blocker (ARB) if patient was microalbuminuria positive, retinal examination, and foot examination. The clinical indicators included nine items: FBS (80–130 mg/dL), blood pressure (<140/90 mmHg), HbA1c (<7%), LDL (<100 mg/dL), hospitalization due to acute diabetes complications, diabetic retinopathy, diabetic nephropathy, foot wound, and amputation of toes, foot, or leg. Descriptive statistics were used to assess the quality of diabetes care.

### Step 3: focus group

The inclusion of focus groups in the final step aimed to elucidate awareness of care performance by seeking feedback from frontline workers concerning ways to improve service. The goal was to identify factors that were supportive for diabetes care as well as factors that posed obstacles to providing optimal diabetes care. Before the focus group met, the descriptive results of the study were presented to participants, which informed them about the overall performance of the care. The discussion followed, with topics including important factors that influenced the success of diabetes care, strengths and limitations of the current approach to care, how the hospital supported this service, and suggestions for improving the approach to care. Similar to Step 1, the focus group guide was reviewed by two experts to ensure validity of the guide. The focus group discussion was held in a meeting room of the district health network center and lasted approximately 1.5–2 h. Participants were given research information and a signed consent form prior to the meeting.

Interviews and discussions for Steps 1 and 3 were audio recorded and transcribed verbatim, followed by content analysis using a quality of care framework that centered on three key areas: structure of care, care process, and outcome (Campbell *et al.*, 2000). In accordance with this framework, thematic categories were stipulated prior to conducting data analysis. All transcripts were read thoroughly. Coding of the qualitative data was performed. Portions of the dialogue where ideas were proposed or concerns were expressed regarding diabetic care were labeled by words, phrases, or sentences. This step aimed to parse the text data. All codes were reviewed and aggregated in order to identify common themes. Data from interviews and focus groups were analyzed separately. Participants did mention obstacles to delivering care, without being explicitly asked this question in the interview. Results from both sources were combined when reporting on the challenges facing optimization of diabetic care, since there was a cross-cutting connection between them (Green and Thorogood, 2004).

## Findings

### Study participants

Ten full-time primary care practitioners working in a SHPH diabetes clinic participated in the study. In step 1, five participants were interviewed face-to-face whereas another five were interviewed via telephone. In step 3, two focus groups were created: one group had six practitioners and the second group had four practitioners (the same participants who were previously interviewed). All were female and included five registered nurses, four nurse practitioners, and one public health technical officer. The average age of participants was 40.4 ± 4.58 years old with an average work experience of 9.3 years in a diabetes clinic.

### Diabetes care process

**Figure 1.**
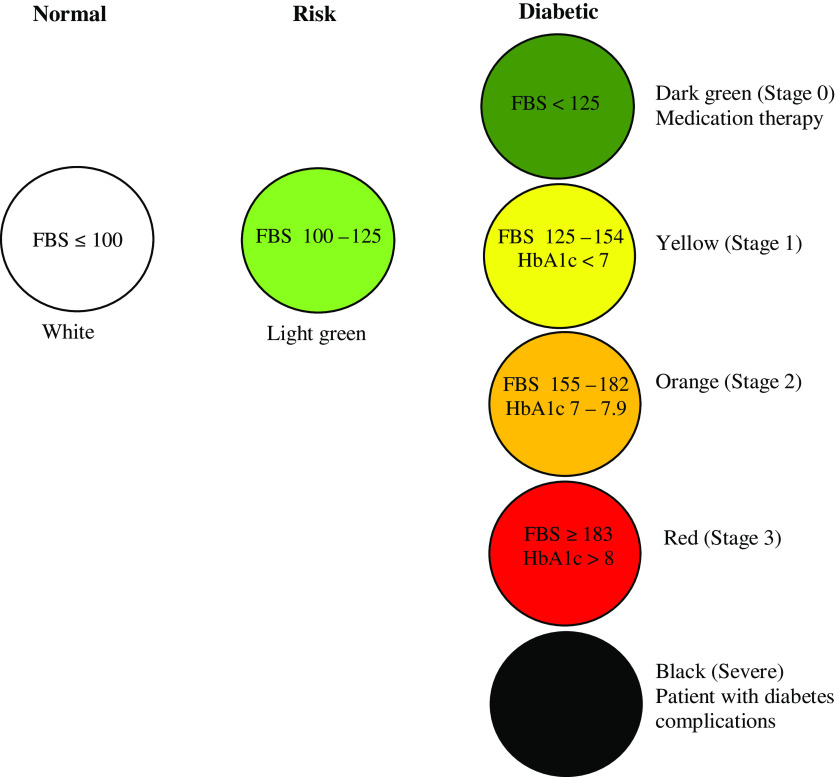
Vichai’s 7 color balls model (Thianthawon, 2013).

**Figure 2.**
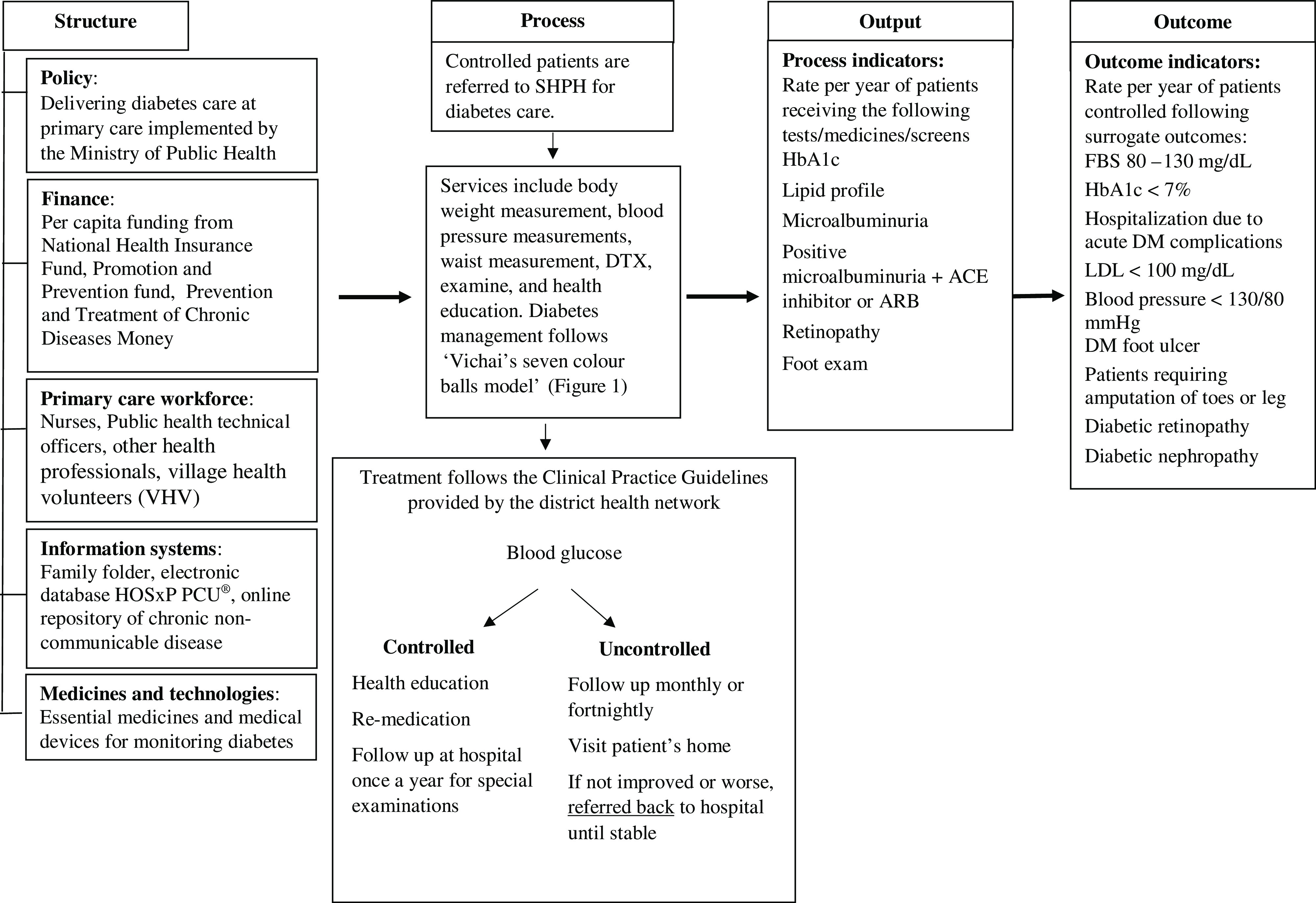
Diabetes care in SHPH.

District health network boards established the diabetes care process at a SHPH, which was implemented in accordance with district clinical practice guidelines. The care process consisted of multiple steps and involved collaboration between various health care providers from a designated hospital and its associated SHPH. Patients were screened for several vital surrogate indicators and examined by a nurse or public health technical officer, followed by dispensation of medication. Patients were classified into seven types according to ‘Vichai’s seven color balls model’ (Figure 1) based on their clinical outcomes (Thianthawon, 2013). Patients with well-controlled diabetes were placed in the green group; these patients were fast tracked for receipt of service (shorter waiting times). Patients in the green and yellow groups were given bimonthly follow-up appointments. Patients in the red and orange groups were those whose outcomes were poorly controlled, thus requiring them to have a follow-up appointment every month. However, if patients in the red and orange groups did not demonstrate improvement they were then monitored every two weeks. In the groups where patients’ diabetes was not well controlled, individuals were assigned a variety of activities to engage in. These included exercise, meditation, and games intended to promote physical activity (Figure 2).

‘We used "Vichai’s seven colour balls model" for grouping patients. Most of the patients were in the green and yellow group … For the patients with very high blood glucose, I would give them some additional advice during the examination’. (Interview 9)

If necessary, the red group patients would be referred back to the hospital when diabetes was found to be poorly controlled at two consecutive appointments or if adverse events such as hypo- and hyperglycaemia had occurred. However, all patients were sent to the hospital annually to undergo other essential laboratory examinations.

‘If the patients could control their blood glucose level, we would refer them once a year … but if FBS was above 160 mg/dl at two consecutive follow-up appointments, they would be referred to the hospital to adjust their treatment’. (Interview 8)

In addition, a primary care team from the SHPH had the option to visit the red group patients who were experiencing complications, in order to identify other possible causes for a patient’s uncontrolled symptoms and adverse events.

‘Most of the home visits were made to patients with chronic wounds or other complications’. (Interview 4)

## Quality of diabetes care

Of the 1885 patients registered in the electronic database, 349 were eligible for inclusion. General characteristics of included patients are shown in Table 1. Table 2 presents a summary of the overall quality of diabetes care. Based on the process indicators, over 80.0% of patients received four essential services: HbA1c monitoring, microalbuminuria monitoring, retinopathy screening, and foot examination. Approximately one-third of patients (34.2%) with microalbuminuria received an ACE inhibitor or ARB. Lipid profile monitoring received a value of 0.0% because patients were screened for LDL and triglyceride levels only, not the full lipid panel.

**Table 1.** Demographics of eligible patients

Variable	*n* (%)
Gender	
Male	81 (23.2)
Female	268 (76.8)
Age (year) mean ± SD	62.54 ± 9.93
Duration of diabetes (year) mean ± SD	7.55 ± 3.24
Health insurance scheme	
UCS	339 (97.1)
CSMBS	10 (2.9)
Comorbid diseases	
Hypertension	147 (42.1)
Dyslipidemia	7 (2.0)
Cerebrovascular diseases	2(0.6)

UCS = Universal Health Coverage Scheme (UCS); CSMBS = Civil Servant Medical Benefit Scheme.

**Table 2.** Quality of diabetes care

	*n* (%)
Process indicators	
1. HbA1c^a^	308 (88.3)
2. Lipid profile^*^^,a^	0 (0.0)
3. Microalbuminuria^a^	285 (81.7)
4. Positive microalbuminuria, administered ACE inhibitor, or ARB^b^	64 (34.2)
5. Retinopathy screening^a^	318 (91.1)
6. Foot examination^a^	334 (95.7)
Outcome indicators	
1. FBS 80–130 mg/dl^c^	136 (34.5)
2. HbA1c < 7%^d^	89 (28.9)
3. Hospitalization due to acute DM complications^a^	3 (0.9)
4. LDL < 100 mg/dl^**^^,e^	110 (34.8)
5. Blood pressure < 130/80 mmHg^a^	252 (72.2)
6. Having a DM foot ulcer^a^	12 (3.4)
7. Requiring their toes, foot, or leg cut off^a^	0 (0.0)
8. Diabetic retinopathy^f^	28 (8.8)
9. Diabetic nephropathy^a^	58 (16.6)

Denominator for each indicator: ^a^349, ^b^187, ^c^394, ^d^308, ^e^316, ^f^318.

* Lipid profile = Cholesterol, LDL, HDL, and Triglyceride.

** Used data of the previous fiscal year (2016/2017).

The percentage of patients who had controlled LDL, FBS, and HbA1c <7% was quite low, (34.8, 34.5, and 28.9% respectively), while blood pressure was controlled better (72.2%). Other occurrences of reported diabetic complications included foot ulcer (3.4%), diabetic retinopathy (8.8%), and diabetic nephropathy (16.6%).

## Challenges of diabetes care in primary care

While evidence for support was documented in the interviews and focus group discussion (FGDs), a number of challenges regarding delivery of optimal diabetes care via SHPH were identified. Review of the qualitative study data revealed that significant limitations to delivering care were related to available health service resources, delivery of services, and patient factors.

## Health service resources

Five issues related to resources that support diabetes care offered by SHPH were mentioned frequently, and included policy issues, financial support, availability of medicines and technologies, sufficient primary care workforce, and efficient information systems.

### Policy

Most participants supported the primary care policy to follow-up diabetic patients at SHPH because it benefitted patients, especially in terms of reducing transportation costs incurred by the hospital, with the SHPH being more accessible. Participants also perceived that patients were satisfied with this service, and emphasized that this approach could help reduce hospital congestion. However, some participants did not agree with this policy because they sensed that some patients were sceptical about service potential related to primary care – namely that patients were more confident in doctors than primary care practitioners.

‘Patients would experience a reduction in costs for travel to a medical care facility; this would also reduce hospital congestion. The SHPH staff could provide full care for patients at their facility’. (Interview 8)

‘I did not agree [with the policy] because, although patients were happy to be referred to the primary care unit that was closer to home, they [patients] were more confident in the care provided by a doctor than by the nurse’. (Interview 6)

In addition, some participants reported that this policy had impacted the workload of primary care practitioners and also increased the financial burden on their health services facility.

‘Personally, I think this policy has placed an added burden onto us. Still, I do follow this policy because patients and others appreciate it. If I could rate my approval of this policy, I would give it a six out of ten. I wouldn’t give it a full score of ten because I’m tired’. (Interview 10)

### Finance

In terms of financial support, diabetic care services at the SHPH received a budget from three governmental sources: per capita funding from the National Health Insurance Organisation [NHSO], a budget for promotion and prevention, and a budget for prevention and treatment of chronic diseases. The first was to procure medicines and supplies, the second for health promotion and prevention activities. The third was to fund screening for medical complications and behavior modification for those whom were already diagnosed. Although each source was intended to support a different aspect of care, all sources of funding could be pooled to optimize delivery of diabetic care.

‘The budget for providing diabetic care came from the medication budget [NHSO], health promotion and prevention, and the budget for prevention and treatment of chronic diseases. The budget for prevention and treatment of chronic diseases represented a special project that required the hospital team to submit a proposal to NHSO’. (Interview 7)

In addition, diabetes care provided at the SHPH was supported financially by local authorities such as the Sub-district Administration Organization (SAO). This support allowed for the purchase of screening test kits, while donations from local entrepreneurs and the public provided quick meals/snacks for patients at their follow-up appointments.

‘SAO supports the purchase of screening test kits’. (Interview 7)

Participants commented that the budget for procurement of medications was limited. Since the number of diabetic patients receiving sub-district primary care had been increasing, this has subsequently led to an increase in medication costs. They suggested that the budget for chronic medication should be expanded due to the increased number of patients.

‘When we ordered more medicine from the hospital, we were asked “Why does sub-district primary care have the highest medical cost per capita?” Therefore, I feel that I have to carefully control the number of medications I prescribe, while also reducing other costs’. (Interview 2)

‘The budget for medicine was too little’. (focus group 7)

### Availability of medicines and technologies

Participants reported that the medical equipment necessary to provide diabetes care was adequate, particularly for equipment used within the primary care center. This included digital blood pressure measuring devices, blood glucose meters, and scales. Each of these items is calibrated every month. Nonetheless, one problem reported by health care personnel was that the blood sugar strips provided by the hospital were a newer version that was not compatible with the meter being used to read the strips. Participants suggested that the hospital should confirm the availability of specific medical supplies prior to proceeding with procurement.

‘The SAO purchased blood glucose meters for us five or six years ago, but currently the strips used with that specific meter model were out of stock’. (focus group 7)

### Primary care workforce

The number of providers delivering diabetes care at the SHPHs ranged from four to six people. This number included nurses, public health technical officers, and other health assistants. The nurses and public health technical officers played important roles in providing medical treatment, health prevention, health promotion, and dispensing medicine. At some SHPHs, other health staffs (such as the public health officer or dental nurse) were permitted to dispense medication. Village health volunteers (VHV) assisted in measuring blood glucose (using a finger prick test device), monitoring blood pressure, waist dimensions, and body weight. They also provided basic health education to groups of patients.

One concern that was raised was that the number of staff and their competency in diabetes care was inadequate. Participants stated that diabetes care offered in primary care substantially increased their workload, and that the current number of available staff was not sufficient to cover the requested caseload. Therefore, they suggested recruiting health care providers from the hospital to assist with providing this service. Moreover, participants opined that the training of VHVs needed to be enhanced, to ensure their knowledge and increase confidence in providing basic health education to patients.

‘Nurses do everything in the primary care. Because of the significant increase in the workload, I need to have additional staff’. (focus group 3)

‘I would like to have a nutritionist and a pharmacist provide assistance with this service. I need a team to deliver a high level of support to the patients’. (focus group 1)

‘I would like other care facility staff to understand more about diabetes, so that if I was busy they would be able to provide service and advice to the patients’. (Interview 1)

The VHVs are a potential source of additional support in the care of uncontrolled diabetic patients within the community. One interesting proposal was to pair one VHV with one uncontrolled patient. The VHV’s role would be to encourage the patient to modify their health habits in order to achieve control of blood glucose levels. It was recommended that successful VHVs would be compensated for taking on the additional responsibilities required to perform this service.

‘Assign one VHV to one patient age 65 years or older who has high blood glucose levels. The VHV who could help the patient control their blood glucose level would be rewarded’. (focus group 10)

### Information system

All services provided were recorded manually in the patient’s family folder and electronically in HOSxP PCU^®^ (an electronic health database system widely used in Thailand). Data was then uploaded to the provincial online database, the ‘repository of chronic non-communicable disease’. However, errors were occasionally found regarding the recording of patients’ information in this online repository. In one instance, a non-diabetic patient had been registered as having diabetes. Thus, information entered into the database requires some method for verification in order to ensure the accuracy of the recorded data. Participants also commented that uploading data to the online repository was yet another time-consuming task assigned to the primary care staff.

‘Regarding the patient information system, there were some non-diabetic people recorded as being diabetic in the online database. Someone needs to recheck it’. (focus group 6)

## Service delivery

Participants recommended that the hospital and SHPH teams work collaboratively on home visits to a patient. The goal of this service has been to help diabetic patients control their blood glucose levels in order to live a normal, healthier life. The collaborative team should set goals and design an action plan for each visit; only diabetes-related health providers are needed for home visits. Given that the number of available care staff is often limited, such an approach would mean that it would not be necessary for all multidisciplinary team members to attend every home visit.

‘I would like the team to oversee physical activities, home visits, and diet control, not simply to conduct a physical examination and dispense medicines’. (focus group 7)

## Patient factors

The clinical outcomes shown in Table 2 indicate a limited ability to control blood glucose levels and HbA1c. Participants suggested that this might reflect an individual patient’s lack of awareness of their health status, but could also be related to the nature of the patient’s employment. Participants indicated that patients routinely received appropriate health education at the primary care clinic, but acknowledged that it may be necessary to elevate a patient’s understanding as to what is required for them to maintain a healthy lifestyle.

‘The important thing is that the patient should realise that they must take charge of their own health. Patients are provided with a lot of health-related information’. (focus group 9)

Regarding a patient’s occupation, for example, some elderly people still work to support their family, while some work as rubber tappers. Since these workers typically work late at night, they may be inclined to drink energy drinks, which have a high sugar content, to remain alert and awake. This represents a difficult obstacle for health care providers to overcome, perhaps requiring assistance from other stakeholders.

‘Patients often have long working hours, such as during the rice farming season. They may simultaneously work at more than one job - rubber tapper, raising cattle/buffalo - and drink energy drinks to stay awake’. (focus group 9)

## Discussion

This study has presented an overview of diabetes care delivered via primary care systems in Thailand, and some insights have been revealed. The process of care has been well established, and is supported by the collaborative work that occurs between the hospital and the SHPH. The quality of SHPH diabetes care was estimated to be good with regard to only five indicators (four process indicators and one outcome indicator). A number of findings provide support for such a collaborative effort, but potential challenges to delivering care in this way have been identified. Opinions on the current policy to follow diabetic patients at SHPH were mixed, some being positive and others negative. There was a request to expand the budget for medicine and to check the specifications of the required medical devices prior to purchasing equipment or consumables such as blood sugar test strips. One major concern among providers was whether there were a sufficient number of clinicians and non-clinicians in the workforce. In addition, competency of some of the providers in diabetes care was questioned, and the increased workload for providers had been noted as inappropriate.

The structure of a health care system includes policy, health care workforce, financing, access to essential medicines, and health information systems (World Health Organization, 2010). The results of the current study indicate that SHPH diabetes care at this study site aligns with recommendations by WHO. The policy for diabetes care delivered via primary care units helps increase accessibility to the service, particular in remote areas, and may reduce the cost of transporting patients to the hospital. This approach may subsequently improve quality of diabetes care at the individual level, since accessibility to service has been expanded (Campbell *et al.*, 2000; Mosimah and Battle-Fisher, 2017).

A previous study reported that diabetes care at SHPHs may be more effective in some regions of the country compared to others (Srivanichakorn, 2007). There could be a variety of reasons for such a finding, such as comparatively more support from the local district health network, the strength of the workforce, and better coordination and involvement from the local community (Srivanichakorn, 2009). The care presented in this study is from one of the well-established models of collaborative work provided by the district health network. A mixture of clinic-based and proactive activities has been included in SHPH diabetes care. ‘Vichai’s seven color balls model’ is a concept that facilitates the classification of patients in order to provide appropriate care based on the severity of their diabetes, as recommended by the Ministry of Public Health (Thianthawon, 2013). This concept has been also used in other district health networks (Tossanoot and Sirikamonsathian, 2016; Sadtrakulwatana, 2018).

The American Diabetes Association recommends that health care providers conduct appropriate health examinations for diabetic patients, including assessment of HbA1c levels, lipid profiles, nephropathy, and retinopathy screening (American Diabetes Association, 2016). NHSO also encourages all SHPH to follow these guidelines to ensure quality of care (National Health Security Office, 2017). Primary care units in Hong Kong also screen diabetic patients for smoking status (Wong *et al.*, 2012). A previously published systematic review reported a negative association between process indicators measuring numbers of tests and clinical outcomes (Sidorenkov *et al.*, 2011), but it is clear that both leading associations in diabetes and policy makers have observed that the process indicators are important for ensuring the quality of diabetes care. Our study found that ACE inhibitors or ARB were not routinely given to patients who tested positive for microalbuminuria. This may reflect the physician’s lack of confidence in the results of a dipstick test (Pithaksa and Prasannit, 2015) since urinary albumin excretion can also be affected by other factors and conditions such as exercising in the 24 h prior to testing, infection, and fever (American Diabetes Association, 2016). In contrast to findings from previous studies (Parinyasakulwong, 2009; Rangsin *et al.*, 2012), the proportion of patients who had lipid profile data was zero because the current study site followed the WHO/ISH risk prediction charts which instead emphasizes cholesterol monitoring (World Health Organization, 2014). The performance of outcome indicators was very low, with one exception being the monitoring and control of blood pressure. This is because patients with normal blood pressure would be referred to SHPH.

Similar to other developing countries where the shortage of doctors is an issue, non-physician clinicians will be key for managing chronic diseases in the primary care setting, but these personnel need appropriate training in order for them to gain patients’ trust in the quality of care they provide (Beaglehole *et al.*, 2008). The health workforce for diabetes care at SHPH is likely to be inadequate due to the heavy workload, limited number of staff, and lack of competently trained individuals, a finding that is consistent with previous studies (Briggs *et al.*, 2007; Koshakri, 2008; Srivanichakorn, 2009; Tangcharoensathien *et al.*, 2016). In Thailand, all non-physician clinicians working in diabetes care are trained in appropriate programs and the quality of care is monitored regularly. A strong health workforce is viewed as an important driver of the health system, as suggested in the WHO’s six ‘building blocks’ (World Health Organization, 2010). Adequate health workforce, both in terms of the number and competency of providers, are believed to underlie positive outcomes. Another barrier to providing optimal diabetes care in the community may relate to communication skills. Increasing the fluency of staff and VHV in speaking the local dialect could overcome this barrier to delivering care. (Brez *et al.*, 2009; Elliott *et al.*, 2011).

The budgets used for diabetes care at SHPH were derived from several sources with different intended purposes (Srithamrongsawat *et al.*, 2011). The Ministry of Public Health allocates per capita funding to the hospital specifically for purchase of medication and medical supplies. Together with the support from SAO, the budget is sufficient for purchasing blood sugar test devices. However, as mentioned before, there was at one instance where incompatibility between the meter being used and strip supplied posed a significant problem; this situation was also reported in another study (Koshakri, 2008). Participants in our study suggested that the hospital confirm specific features of medical supplies prior to proceeding with procurement. The current study found that the allocated budget was adequate for delivering diabetes care, as was also reported by a previous study (Koshakri, 2008). Nevertheless, one persistent problem was a significant delay in obtaining approval for funding.

Trustworthy health information is important for making health care decisions at health care facilities. The information system used in this study site is a combination of paper- (patient registry, family folder) and electronic-based (HOSxP PCU^®^ and an online repository diabetes database) systems. The time required to record data electronically was reported as imposing a significant impact on staff workload (Nguyen *et al.*, 2014). In our study, participants had to spend additional time uploading data to the online repository, which adds to the workload burden for participants. It is likely that primary care practitioners in this study have twice the workload for record keeping, as they manage their own information systems and contribute their clinical information to the shared database.

## Strengths and limitations

This study provides a review of the quality of diabetes care in primary care facilities based on the quality indicators previously established by national policy makers. Since the structure indicator is not included in this series of indicators, we applied a qualitative approach to reveal problems that had not previously been identified. In the current study, the diabetes care process was derived from the experience of true operational workers. Here, we used the same participants in the interview and the focus groups. A previous review recommended that the use of the same participants in the sequential qualitative mixed method is permissible if the data derived from each phase need to be linked (Morse, 2010). Since two interview modes were used based on convenience for the participant, the limitations of this approach should be considered. For example, while face-to-face interviews may help to focus attention on the questions and the interviewer, it could create social pressure to provide a specific, expected response. Participants interviewed via telephone could potentially feel more relaxed in this regard, but they might also be more easily distracted by their environment (Oltmann, 2016). Such limitations should therefore be considered, as they could affect interpretation of the data. The inclusion of a focus group as a final step in our study provided an opportunity for the primary care team to assess the quality of their performance. Such an approach will very likely help teams improve the quality of care in their health network. However, it is important to note that this study was conducted in one district health network, thus the generalizability of findings may be limited since the process of care may differ between localities.

## Recommendations to policy and practice

The diabetes care process employed at this study site is comprehensive, containing multiple components such as eligibility criteria for patient transition, practice guidelines, health education, and home visits. This can certainly serve as a model for other health networks to follow, especially in locations where diabetes care has not yet been successfully established. As reported in previous studies, current study participants indicated that the primary care work force is a crucial component of an effective system (Srivanichakorn *et al.*, 2015; Tangcharoensathien *et al.*, 2016). The family doctor is a key leader of the family care team, yet a previous survey reported that only about 5% of family doctors actually work in primary care units (Suphapon *et al.*, 2013). This reinforces the idea that this type of care does not depend exclusively on the doctor. Consequently, the burden of diabetes management in SHPH has been shifted to non-physician professionals. The problem regarding an experienced workforce should not be overlooked. Not only is an increase in the number of primary care practitioners needed, a reasonable workload for each worker and fair remuneration are also compulsory. This study also revealed coordinated efforts between local entrepreneurs and the primary care service, as the entrepreneurs supported the effort by donating food for patients at the clinic. It may in the future be possible for the government to garner more involvement from public and/or private entities by proposing a tax benefit for businesses that make donations.

## Conclusion

The diabetes care process in primary care includes multiple steps and involves collaboration between various health care providers from a hospital and a SHPH. Only four process indicators and one outcome have been achieved, indicating a need for improving this approach to providing care. Challenges inherent in attempting to deliver this type of service relates to the resources available to the health service, delivery of services, and other patient-related factors. Additional support is required to strengthen diabetes care in primary care settings nationwide.
